# IFN type I and II induce BAFF secretion from human decidual stromal cells

**DOI:** 10.1038/srep39904

**Published:** 2017-01-06

**Authors:** Anna-Carin Lundell, Inger Nordström, Kerstin Andersson, Christina Lundqvist, Esbjörn Telemo, Silvia Nava, Helen Kaipe, Anna Rudin

**Affiliations:** 1 Department of Rheumatology and Inflammation Research, Institute of Medicine, Sahlgrenska Academy, University of Gothenburg, Sweden; 2 Division of Therapeutic Immunology, Department of Laboratory Medicine, Karolinska Institutet, Stockholm, Sweden; 3 Clinical Immunology and Transfusion Medicine, Karolinska University Hospital Huddinge, Stockholm, Sweden

## Abstract

B cell activating factor (BAFF) is a critical cytokine for maturation of immature B cells. In murine lymph nodes, BAFF is mainly produced by podoplanin-expressing stromal cells. We have previously shown that circulating BAFF levels are maximal at birth, and that farmers’ children exhibit higher BAFF levels in cord blood than non-farmers’ children. Here, we sought to investigate whether maternal-derived decidual stromal cells from placenta secrete BAFF and examine what factors could stimulate this production. We found that podoplanin is expressed in decidua basalis and in the underlying villous tissue as well as on isolated maternal-derived decidual stromal cells. Decidual stromal cells produced BAFF when stimulated with IFN-γ and IFN-α, and NK cells and NK-T-like cells competent of IFN-γ production were isolated from the decidua. Finally, B cells at different maturational stages are present in decidua and all expressed BAFF-R, while stromal cells did not. These findings suggest that decidual stromal cells are a cellular source of BAFF for B cells present in decidua during pregnancy.

B cell activating factor (BAFF) is critical for survival and differentiation of immature transitional B cells into mature naïve cells. BAFF-deficient mice present with normal B cell development up to the transitional stage but additional maturation in the spleen is hampered^1^,^2^. These mice also exhibit reduced antibody titers in response to both T-dependent and T-independent antigens^1^. Human BAFF-R-deficiency resembles the murine phenotype by arrested B cell maturity at the stage of transitional B cells and reduction in the numbers of all subsequent B cell maturational stages^3^. Although BAFF is required for B cell homeostasis and function, the cellular source(s) of BAFF remains to be explored further. Innate immune cells and epithelial cells produce BAFF in response to IFN type I (IFN-α) and type II (IFN-γ) *in vitro*^4^,^5^,^6^,^7^, but their ability to produce physiologically relevant BAFF levels *in vivo* has not been determined. Results from mouse models instead indicate that stromal cells are the main source of BAFF to support normal B cell homeostasis *in vivo*^2^.

Murine lymph nodes are comprised of a heterogeneous stromal network that can be distinguished by expression of podoplanin and CD31, e.g. fibroblastic reticular cells, follicular dendritic cells as well as lymphatic and blood endothelial cells^8^,^9^,^10^. In mice, podoplanin is crucial for the development of the lung and deep lymphatics, as podoplanin knock-out animals die soon after birth as a result of respiratory failure and generalized lymphedema^11^. An endogenous receptor of podoplanin is C-type lectin-like receptor-2 (CLEC-2), which is primarily expressed on platelets and dendritic cells^12^,^13^. On dendritic cells, CLEC-2 functions by facilitating efficient motility of activated dendritic cells along stromal podoplanin-expressing surfaces^13^. The murine stromal network also includes intertwined fibers that express the ER-TR7 antigen, which is detected by Erasmus University Rotterdam thymic reticulum antibody 7^10^,^14^. Although ER-TR7 is localized in close proximity to podoplanin, and is used as a marker to identify fibroblastic reticular cells^15^, the antigen of ER-TR7 has yet to be determined. The fibroblastic reticular stromal cell network has been shown to function as a conduit system for immunocompetent cells to reach appropriate sites in order to cause immune reactions^16^. Over the past decade, discoveries relating to stromal cells have promoted these cells from bystanders to key players in immune responses^8^,^13^,^17^,^18^. Podoplanin-expressing follicular reticular cells were recently shown to be the primary cellular source for local BAFF in murine lymph nodes^19^. A similar podoplanin-positive reticular stromal cell network is also present in the T cell zone in human lymphoid tissue^10^, but if this network possesses the same functions as demonstrated in mice remains to be examined.

Longitudinal data regarding blood BAFF concentrations in early childhood have been lacking. We recently showed that BAFF levels in plasma were maximal at birth and that newborn children have higher levels than their mothers, the latter a finding also observed by others^20^,^21^,^22^. In the same study we also demonstrated that farmers’ children had elevated BAFF levels at birth compared to non-farmers’ children, and that higher neonatal BAFF levels were associated with an accelerated B cell maturation later in childhood^20^. Yet, both origin for BAFF in the fetal circulation and BAFF-inducing factors associated with a farming environment are unknown.

The placental basal plate (Fig. 1j) is an area of direct contact between maternal and fetal tissues, which includes maternal-derived decidua basalis and fetal-derived villi that are bathed in the circulating maternal blood. The outermost layer of the villi consists of syncytiotrophoblasts that form a continuous epithelium generated and maintained by the underlying cytotrophoblast cells. The latter layer of cells is supported by a basement membrane that separates it from the villous core, including stroma, capillaries and leukocytes (Fig. 1k). Decidual tissue is composed of stromal cells as well as different leukocyte subsets, including T cells, NK cells, NK-T cells and B cells^23^,^24^,^25^,^26^. B cells are found in the decidua during the whole pregnancy^26^,^27^, and it has been shown that fetal membranes have the ability to attract B cells in experimental settings^28^. Expression of podoplanin has been demonstrated in human placenta, mainly in the villous stroma, throughout gestation and BAFF is expressed intracellularly by decidual stromal cells^25^. Still, it is not known whether decidual stromal cells secrete BAFF and which stimuli that trigger its production. Although circulating B cell subsets, including transitional, mature/naïve, memory and regulatory B cells, have been characterized from late pregnancy to post-partum^29^, it remains to be elucidated if B cells at different maturational stages are present in decidua and if they express BAFF-R.

In the present study we explored the role of decidual stromal cells as a possible source of BAFF. In placentas from full term normal pregnancies, we demonstrate that podoplanin is expressed in decidua basalis and in the underlying villous tissue as well as on isolated maternal-derived decidual stromal cells. We also found that decidual stromal cells produced BAFF when stimulated with IFN-γ and IFN-α, while cord blood mononuclear cells did not. NK cells as well as NK-T-like cells competent of providing IFN-γ to the stromal cells, and BAFF-R-expressing B cells at different maturational stages were present in the decidua. Thus, decidual stromal cells could be a cellular source of BAFF for decidual B cells as well as for BAFF in the fetal circulation, which could explain the environmental effects on BAFF levels in neonates.

## Materials and Methods

### Placenta and cord blood

Human term placentas and umbilical cord blood samples were obtained from unselected healthy vaginal deliveries at Mölndal delivery unit, the Sahlgrenska University Hospital (Göteborg, Sweden). All mothers were given oral information, and gave oral consent to participate in the study. According to the Swedish law (2003:460, §4 and §13) ethical approval was not needed as no personal information or identity was recorded and as placentas are normally discarded after delivery. For some of the experiments regarding isolation of decidual stromal cells, i.e. phenotypic characterization and measurement of BAFF secretion, term placentas were obtained from healthy mothers after cesarean section at Huddinge delivery unit, Karolinska University Hospital (Stockholm, Sweden) (Figs 2 and 3 and Supplementary Fig. S2 and S4, respectively). In that case personal information and identity was recorded and ethical approval was thus obtained from the Karolinska institutional ethical review board (2009/418-31/4 and 2010/2061-32). All experiments were carried out in accordance with the approved guidelines and regulations.

### Isolation and expansion of stromal cells from decidua basalis and parietalis

**Figure 1:**
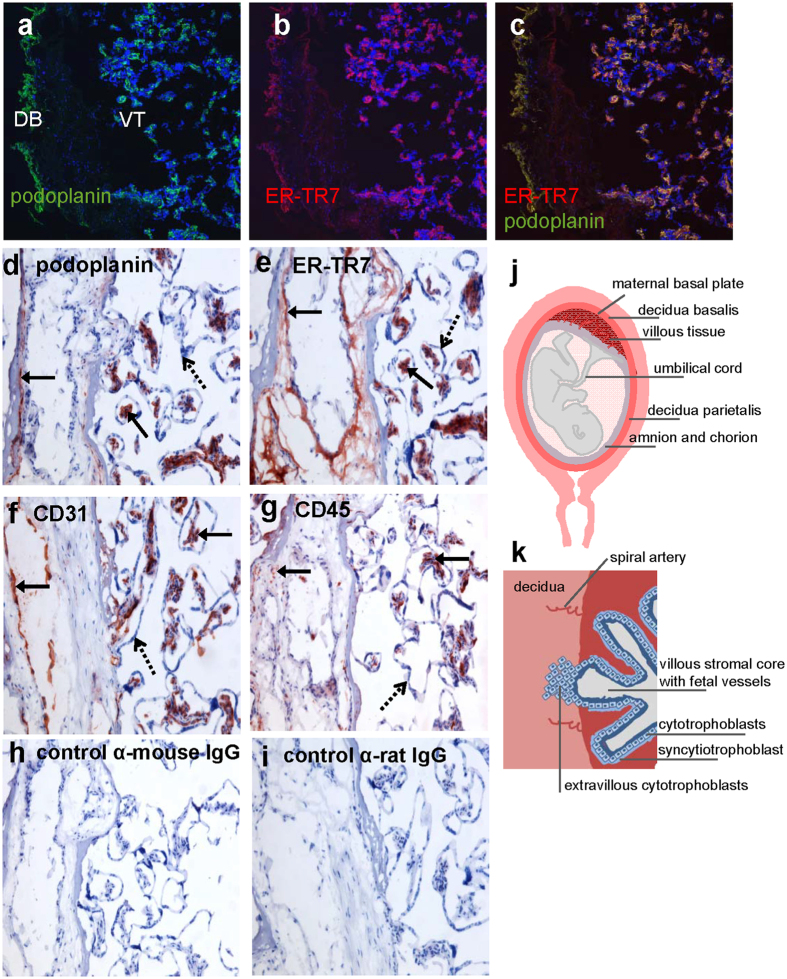
Podoplanin and ER-TR7 expression in placental tissue. Immunofluorescence staining of podoplanin **(a)**, ER-TR7 **(b)** and merge of the two markers **(c)** in decidua basalis (DB) and in the underlying villous tissue (VT). Podoplanin is depicted in green, ER-TR7 in red and nuclei staining with Hoechst in blue (x10 magnification and scale bar 20 μm, one experiment out of two). Immunohistochemistry images of podoplanin **(d)**, ER-TR7 **(e)**, CD31 **(f)**, CD45 **(g)**, mouse and rat isotype control staining **(h** and **i**, respectively) in decidua basalis and underlying villous tissue (x10 magnification). Schematic illustrations, made by A–C Lundell, of a term placenta that show from which parts tissue and cells were isolated **(j)** and of the maternal-derived decidua and fetal-derived villous tissue **(k)**.

The placenta was moved to a sterile beaker containing PBS (HyClone^TM^, Logan, USA). The maternal basal plate of the placenta was placed upward and small tissue pieces from the cotyledons, including decidua basalis and villous tissue, were cut out with a scalpel (about 10 mm^2^ and 2–3 mm deep). Tissue pieces were also dissected from decidua parietalis, including chorion and amnion, (about 10 mm^2^ and 1 mm deep) as previously described in detail^30^. A schematic drawing of a term placenta is shown in Fig. 1j to illustrate from which parts of the placenta tissue pieces were dissected. Tissue pieces were transferred to petri dishes for further extensive washing in PBS. Next, an equal volume of trypsin/EDTA (Thermo Fisher Scientific, Waltham, USA) was added for 10 minutes at 37° and was then discarded. Tissue pieces were then incubated twice in trypsin/EDTA for 40 min at 37°, and thereafter washed in Dulbecco’s modified Eagle’s low glucose medium (DMEM) (HyClone^TM^) containing 10% fetal bovine serum (FBS, HyClone^TM^) and gentamycin (Sigma-Aldrich, St. Louis, USA) (complete DMEM). The remains of tissues were cut into smaller pieces, and these were spread out and incubated in T182 flasks (VWR, Radnor, USA). Tissue pieces were removed from the flasks when colonies of fibroblast-like cells appeared after about 1 week of culture. When the cells from trypsin-digested tissue pieces were 90–95% confluent, cells were harvested with trypsin/EDTA, washed in complete DMEM, and seeded in new T182 flasks in complete DMEM. The cells were cultured to passage 1, 2 or 3 and frozen slowly in FBS containing 7.5% DMSO (Sigma-Aldrich). The stromal cells isolated from the decidua parietalis was confirmed to be of maternal origin^31^. To induce decidualization, stromal cells (2 × 10^5^ cells/ml) were cultured in 24-well plates in complete DMEM in the presence of 300 nM progesterone (P4; Sigma-Aldrich) and 500 μM 8-bromo-cAMP (Sigma-Aldrich) for 7 days^32^. Half of the culture medium was changed on day 4, and P4 and cAMP were readded. Decidualization was assessed by prolactin secretion. The measurement of prolactin was performed in the laboratory of Clinical Chemistry at the Sahlgrenska University Hospital using the routine accredited method electrogenerated chemiluminescence^33^.

### Immunofluorescence staining and immunohistochemistry

Immunofluorescence staining for detection of podoplanin and ER-TR7 was performed on 5–7-μm-thick frozen tissue sections from the maternal basal plate of the placenta, and on isolated decidual stromal cells attached to glass slides by cytospin centrifugation. Slides were counterstained with Hoechst 34580 (Thermo Fischer Scientific) and mounted with vectashield (Vector Laboratories, Berlingame, USA). Images were obtained by use of a confocal microscope equipped with 405-, 488-, 555- and 639-nm lasers (LSM 700, Zeiss, Oberkochen, Germany). Images were acquired with ZEN software (Zeiss). Discrimination of different cells, such as leukocytes, endothelial cells, epithelial cells and stromal cells, in decidua and villous tissue was examined with immunohistochemistry using ImmPRESS^TM^ according to the manufacturer’s instructions (Vector Laboratories, Burlingame, USA; MP-7402 for mouse antibodies and MP-7444 for rat antibodies). Glucose oxidase was used for quenching of endogenous peroxidase activity. Primary antibodies used are indicated in Table 1. Sections were analyzed using a Leica DMR microscope.

### Isolation of mononuclear cells from decidua and cord blood

Tissue pieces from the basal plate, as described above, and from decidua parietalis, with amnion removed, were placed in PBS with 2 mM EDTA (Media Department, Gothenburg University, Sweden) and cut into smaller pieces. Tissue pieces were then transferred into gentleMACS C tubes (Miltenyi Biotec, Bergisch Gladbach, Germany) containing RPMI-1640 and placed in a gentleMACS Dissociator (m_spleen 04.01 programme, Miltenyi Biotec). Next, 50 μg/ml Liberase TL (Roche Diagnostics, Basel, Switzerland) and 40 μg/ml DNase 1 (Sigma-Aldrich) was added to the dissociated tissue for 30 minutes at 37 °C on an orbital shaker. The digested suspension was then passed through 70-μm, followed by 40-μm cell strainers (Thermo Fisher Scientific and Corning Incorporated, New York, USA, respectively). Lymphoprep^TM^ (Axis-Shield PoC AS, Oslo, Norway) was then used to isolate mononuclear cells from the dissociated decidual tissue as well as from umbilical cord blood.

### Stimulation of decidual stromal cells and mononuclear cells

Decidual stromal cells (2 × 10^5^ cells/ml), cord blood and maternal-derived mononuclear cells (1 × 10^6^ cells/ml) were cultured in flat 24- or 96-well plates. Stromal cell cultures were performed in complete DMEM and mononuclear cell cultures were performed in RPMI 1640 (PAA Laboratories GmbH, Pasching, Austria) supplemented with 10% FBS (HyClone^TM^), gentamicin (50 μg/ml; Sigma-Aldrich) and L-glutamine (1 mM; Sigma-Aldrich) in the presence or absence of IFN-γ (10 ng/ml; R&D systems, Minneapolis, USA), IFN-α (10 ng/ml; PBL Biomedical Laboratories, Piscataway, USA), IL-2 (10 ng/ml; Peprotech, New Jersey, USA), TNF (10 ng/ml; R&D systems), polyinosinic-polycytidylic acid (polyI:C) (10 μg/ml; Sigma-Aldrich), *E. coli* LPS (100 ng/ml; Sigma-Aldrich), imiquimod acetate (10 μg/ml; Sequoia Research products, Pangbourne, UK) or CpG (10 ng/ml; InvivoGen, San Diego, USA) for 48 h in 5% CO_2_ at 37°.

### BAFF ELISA

BAFF concentrations in culture supernatants from decidual stromal cells and cord blood mononuclear cells were determined by human BAFF DuoSet^®^ ELISA (detection range 39.1–2,500 pg/mL) according to the manufacturer’s instructions (R&D Systems).

### Flow cytometry

All antibodies used for characterization of decidual stromal cells, and for identification of decidual T cells, NK cells, NK-T cells, B cells and pDCs are listed in Table 1. To identify living leukocytes, cells were stained with Fixable Viability Dye (eFluor 506 or 780, eBioscience, San Diego, USA). For experiments analyzing intracellular IFN-γ and IFN-α production, isolated decidual mononuclear cells (10^6^/ml) were cultured overnight with or without poly(I:C) together with IL-12 (10 μg/ml and 10 ng/ml (Nordic Biosite, Stockholm, Sweden), respectively). Brefeldin A (5 μg/ml, BD Biosciences, New Jersey, USA) was added for the last 3 hours. After surface staining cells were fixed and permeabilized using Cytofix/Cytoperm™ kit (BD Biosciences). Antibodies used for detection of IFN-γ and IFN-α are listed in Table 1. Samples were acquired in a FACSVerse or FACSCanto II (BD Biosciences) equipped with FACSSuite or FACSDiva software and analyzed with FlowJo software (TreeStar, Ashland, USA).

### Quantitative Polymerase Chain Reaction (qPCR)

The relative levels of BAFF mRNA were measured in decidual stromal cells (2 × 10^5^ cells/ml) cultured in complete DMEM with IFN-γ (10 ng/ml), IFN-α (10 ng/ml), *E. coli* LPS (100 ng/ml) or medium alone for 20 h. The cells were lysed with lysis buffer (Qiagen, Hilden, Germany). Total RNA was extracted using an RNeasy Micro kit (Qiagen) and treated with DNase (Qiagen) to remove genomic DNA. Complementary DNA was prepared in a random hexamer-primed SuperScript (Thermo Fisher Scientific) RT reaction. The mRNA levels were determined by qPCR on an ABI Prism 7500 Sequence Detection System using MicroAmp Optical 96-well reaction plates. Primer-probe pairs were as follows: GAPDH (Hs99999905_m1) and BAFF (Hs00198106_m1). Samples (10 ng of cDNA) were run in duplicate in a 20-μl reaction mix with TaqMan Universal PCR Master Mix using the comparative ΔΔ*C*_*T*_ method of relative quantification to calculate the differences in gene expression between stimulated and control cells. As an endogenous control, GAPDH was used to correct for variations in sample loading. Samples were normalized to medium control set to 1. All qPCR reagents were purchased from Thermo Fisher Scientific.

### Statistics

The D’Agostino and Pearson omnibus normality test were used to assess if the data were normally distributed (GraphPad Prism, San Diego, USA). Data were analyzed by Kruskal-Wallis test followed by Dunn’s multiple comparison test or by Wilcoxon signed-rank test as described in figure legends (GraphPad Prism). A *p* value ≤ 0.05 was regarded as being statistically significant (*p ≤ 0.05, **p ≤ 0.01 and *** *p* ≤ 0.001).

## Results

### Podoplanin and ER-TR7 are expressed in decidua basalis and underlying villous core

In murine lymph nodes, podoplanin-expressing fibroblastic reticular stromal cells are the main local source of BAFF^19^. The lymph node stromal network also includes intertwined fibers that express the ER-TR7 antigen localized in close proximity to podoplanin^14^. Confocal imaging of tissue sections from the maternal basal plate of the placenta was performed to examine podoplanin and ER-TR7 expression in decidua basalis and villous tissue (Fig. 1j). As shown in Fig. 1a,b, podoplanin and ER-TR7 were expressed both in the decidua and in villous tissue. Merged images displayed co-localization of these two markers (Fig. 1c). Similar results were obtained when placental tissue from another donor was examined (Supplementary Fig. S1). Immunohistochemistry was also performed on the placental tissue sections to assess what cells among endothelial cells, leukocytes, stromal cells and epithelial cells that are podoplanin- and ER-TR-7-positive. A section including a blood vessel in the decidua was selected (Fig. 1d–i) and a schematic illustration of the maternal-derived decidua and fetal-derived villous tissue is shown in Fig. 1k. Podoplanin and ER-TR7 staining was observed close to the endothelium in the decidua and the two markers were also clearly expressed in the villous core (Fig. 1d,e, indicated by solid arrows). Endothelial cells were stained with CD31 both in the decidua and in the villous core (Fig. 1f, solid arrows). A scattered pattern of CD45-expressing leukocytes was observed in the decidua, while they were closely gathered within the villous core (Fig. 1g, solid arrows). The outermost layer of the villi composed of the trophoblastic epithelial cells was negative for all markers examined (Fig. 1d–g, dashed arrows). Negative isotype control stainings are shown in Fig. 1h,i. These results indicate that stromal cells express podoplanin and ER-TR7, while endothelial cells, leukocytes and epithelial cells do not. Hence, the placental basal plate, an area of direct contact between maternal and fetal tissues, might contain a stromal network that express podoplanin and ER-TR7 similar to what has been shown for lymph nodes.

### Isolated decidual stromal cells express podoplanin but not ER-TR7

**Figure 2:**
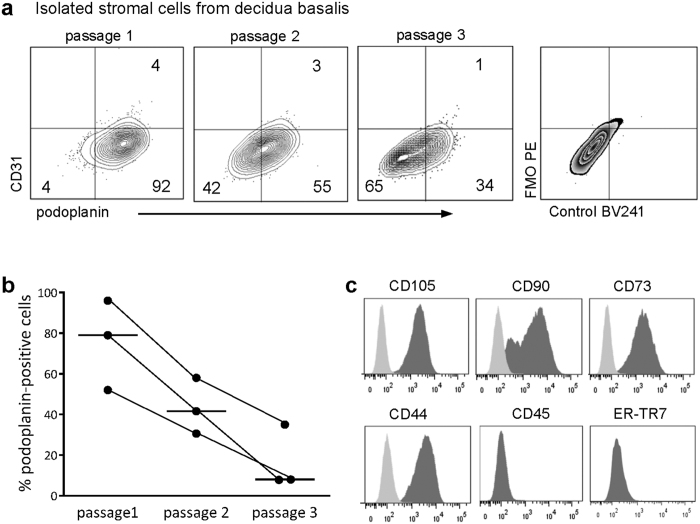
Isolated decidual stromal cells express podoplanin but not ER-TR7. **(a)** Flow cytometry contour plots depict podoplanin and CD31 expression on stromal cells isolated from decidua basalis at different passages. **(b)** The proportion of podoplanin-positive cells at different passages (n = 3). **(c)** Expression of characteristic stroma cell markers on cells isolated from decidua basalis. Specific markers are depicted in dark grey and FMO controls in light grey (one representative experiment out of three).

Next, we examined whether stromal cells isolated from decidua basalis expressed podoplanin and ER-TR7. By the use of flow cytometry we found that a majority of the cells expressed podoplanin, but not the endothelial marker CD31, in the first passage (Fig. 2a,b). However, the proportion of podoplanin-positive cells clearly decreased with time in culture (Fig. 2a,b and Supplementary Fig. S2). The isolated stromal cells displayed a homogenous phenotype with respect to expression of CD105, CD90, CD73 and CD44 that are all markers used to define human stromal cells (Fig. 2c)^34^. All cells were negative for the hematopoietic cell marker CD45 (Fig. 2c) and for CD14 (data not shown), which is in line with the characteristic stroma phenotype. The same phenotype was obtained for stromal cells isolated from the decidua after cesarean section (Supplementary Fig. S2). However, while podoplanin was expressed both in decidual tissue sections as well as on isolated stromal cells, ER-TR7 was not detected on the cultured decidual stromal cells (Fig. 2c and Supplementary Fig. S2). To confirm that the cells are authentic decidual stromal cells, decidualization of the cells was performed with progesterone and cAMP *in vitro*, which resulted in prolactin secretion (Supplementary Fig. S3).

### IFN-γ and IFN-α trigger BAFF production from decidual stromal cells

**Figure 3:**
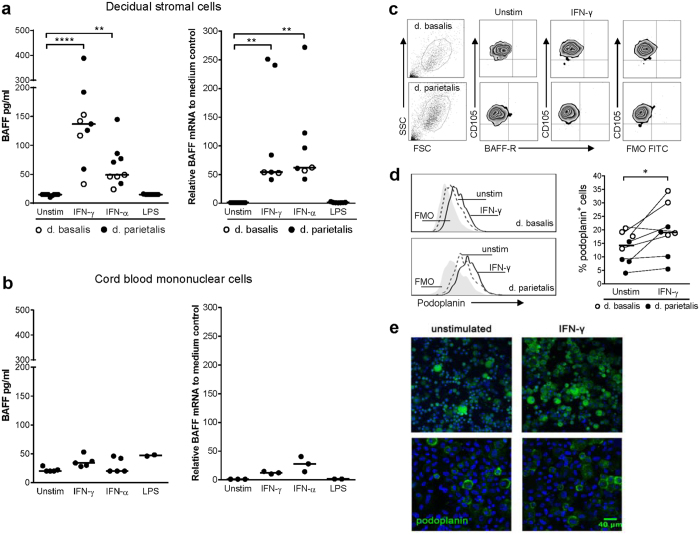
IFN-γ and IFN-α trigger BAFF production from decidual stromal cells. **(a)** BAFF production and relative BAFF mRNA levels to medium control by isolated decidual stromal cells in response to IFN-γ, IFN-α or LPS. **(b)** BAFF production and relative BAFF mRNA levels to medium control by cord blood mononuclear cells in response to IFN-γ, IFN-α or LPS. **(c)** BAFF-R expression on CD105-positive stromal cells isolated from decidua basalis or decidua parietalis after culture in the presence or absence of IFN-γ. **(d)** Histograms depict podoplanin expression on stromal cells isolated from decidua basalis or decidua parietalis and the scatter plot shows the percentage of podoplanin-positive cells after stimulation with IFN-γ or not. **(e)** Immunofluorescence staining of podoplanin on stromal cells isolated from decidua basalis stimulated with IFN-γ or not (podoplanin in green and nuclei staining with Hoechst in blue). Images from one donor in the upper panel are presented with x 10 magnification and images from a second donor in the lower panel are presented with x 25 magnification (scale bar 40 μm). Horizontal bars indicate median. *P ≤ 0.05, **P ≤ 0.01 and ****P ≤ 0.0001, Kruskal-Wallis test followed by Dunn’s multiple comparison test (Fig. 3a) and Wilcoxon signed-rank test (Fig. 3d).

Given the reported role for lymph node podoplanin-positive stromal cells in regulating B cell homeostasis by local production of BAFF^19^, we hypothesized that decidual stromal cells might produce BAFF. To answer this question and to examine if specific stimuli would elicit BAFF production, various cytokines and Toll-like receptor agonists were added to stromal cells isolated from both decidua basalis and parietalis. As shown in Fig. 3a, decidual stromal cells, both from basalis and parietalis, secreted BAFF in response to IFN-γ and IFN-α, but not after stimulation with LPS. No delivery mode-related differences in BAFF secretion were found between cells used from healthy vaginal deliveries and cesarean sections (Supplementary Fig. S4). Moreover, BAFF mRNA levels were clearly upregulated in response to IFN-γ and IFN-α but not to LPS, which supports increased BAFF secretion by these stimuli (Fig. 3a). Stimulation with TNF, IL-2 or Toll-like receptor agonists including Poly I:C (TLR-3), imiquimod (TLR-7) or CpG (TLR-9) did not induce BAFF secretion from stromal cells (data not shown). Contrary to decidual stromal cells, stimulation with IFN-γ and IFN-α or the other stimuli examined did neither trigger BAFF production nor BAFF mRNA expression by cord blood or decidual maternal-derived mononuclear cells (Fig. 3b and data not shown).

As decidual stromal cells produced BAFF, we examined if the main BAFF receptor, i.e. BAFF-R, was expressed on the cell surface. Flow cytometric analysis demonstrated that unstimulated CD105-positive stromal cells were negative for BAFF-R, and stimulation with IFN-γ did not induce BAFF-R or alter CD105 expression (Fig. 3c). These results thus suggest that decidual stromal cells do not secrete BAFF in an autocrine fashion.

Since IFN-γ was the most potent stimuli for BAFF secretion, we also investigated the effects of IFN-γ stimulation on podoplanin expression in decidual stromal cells. The cell cultures had a relatively low baseline expression of podoplanin as cells at later passages (3–4) were used (Fig. 3d). IFN-γ stimulation increased the proportion of podoplanin-positive stromal cells, both from decidua basalis and parietalis (Fig. 3d). These findings were corroborated by confocal imaging on decidual stromal cells from two additional donors. Immunofluorescence revealed that some, but not all, stromal cells from decidua basalis expressed podoplanin and that IFN-γ stimulation increased the number of positive cells (Fig. 3e). Similar results were obtained regarding stromal cells isolated from decidua parietalis (data not shown). Collectively, these data demonstrate that IFN-γ and IFN-α induce BAFF production from decidual stromal cells, which could be a potential cell source for BAFF in the placenta and possibly also for BAFF levels in the fetal circulation.

### BAFF-R expressing B cells at different maturational stages are present in decidua

**Figure 4:**
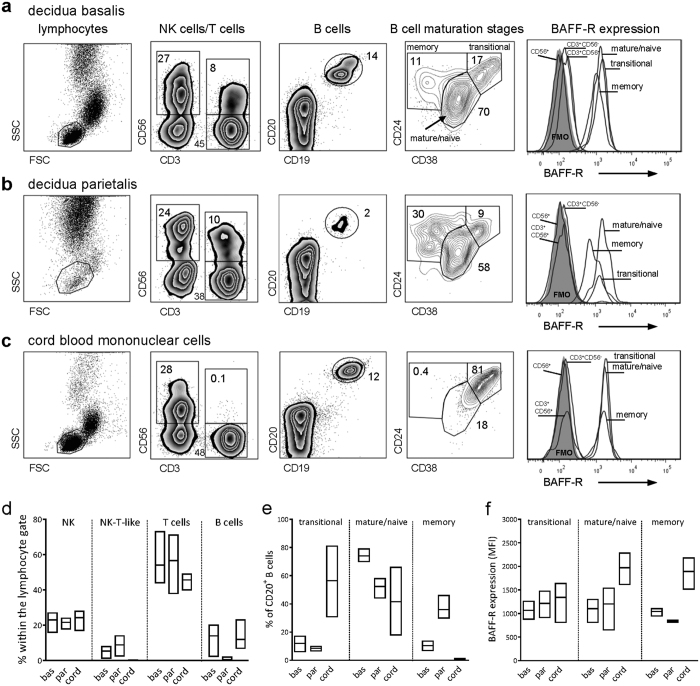
BAFF-R expressing B cells at different maturational stages are present in decidua. To identify lymphocytes, in the first panel to the far left, live cells were gated using a viability dye that marks all dead cells and the lymphocyte gate was then set within CD45-expressing singlet leukocytes. **(a**–**c)** Within lymphocytes, CD56^+^ NK cells, CD3^+^ CD56^neg^ T cells, CD3^+^ CD56^+^ NK-T-like cells and CD19^+^ CD20^+^ B cells were identified (second and third panels). B cell maturational stages were distinguished based on CD24 and CD38 expression (fourth panel). The BAFF-R expression on the different lymphocyte populations is depicted to the far right. **(d)** The proportion of different cell subsets with in the lymphocyte gate and **(e)** the proportion of B cells at different maturational stages isolated from decidua basalis, decidua parietalis and cord blood. **(f)** The mean fluorescence intensity of BAFF on B cells at different maturational stages. Floating bars show minimum to maximum values, and horizontal bar indicates the median (n = 3). Approximately 40.000 cells were collected in the lymphocyte gate for d. basalis and CBMC and 20.000 for d. parietalis.

Although it is known that decidual tissue comprises different leukocyte subsets, including T cells, NK cells, NK-T cells and B cells^23^,^24^,^25^,^26^, it is unknown whether B cells at different maturational stages are present in decidua and if they express BAFF-R. From mother-infant pairs we isolated mononuclear cells from decidua basalis, decidua parietalis, and from cord blood and analyzed the presence of CD56^+^ NK cells, CD3^+^ T cells, CD56^+^ CD3^+^ NK-T-like cells and CD19^+^ CD20^+^ B cells. To distinguish different B cell maturation stages, a combination of CD24 and CD38 was used. First, live cells were gated using a viability dye that marks all dead cells. Next, the lymphocyte gate was set within CD45-expressing leukocytes (Fig. 4). We found clear populations of CD56^+^ NK cells and CD3^+^ T cells in decidua basalis and parietalis as well as in cord blood, while CD56^+^ CD3^+^ NK-T-like cells were only present in decidua (Fig. 4a–d). B cells were present in all three different cell sources, but a higher proportion of B cells within the lymphocyte gate was obtained from decidua basalis compared to parietalis (Fig. 4a–d). Additionally, the decidua comprised B cells at different maturation stages, i.e. immature/transitional, mature/naïve and memory cells, but the fraction of memory B cells was about threefold higher in decidua parietalis compared to basalis (Fig. 4a–c,e). As expected, the majority of B cells in cord blood were of an immature/naive phenotype (Fig. 4a–c,e). Finally, BAFF-R was expressed solely on B cells both in decidua, including all maturation stages, and in cord blood (Fig. 4a–c,f). Flow cytometry results from the other two mother-infant pairs are presented in Supplementary Fig. S5. Thus, since BAFF signaling via BAFF-R is pivotal for differentiation of immature B cells into mature naïve cells, BAFF production by decidual stromal cells could be of importance for local B cell homeostasis during pregnancy.

### NK cells, NK-T-like cells and T cells able to produce IFN-γ are present in decidua basalis

**Figure 5:**
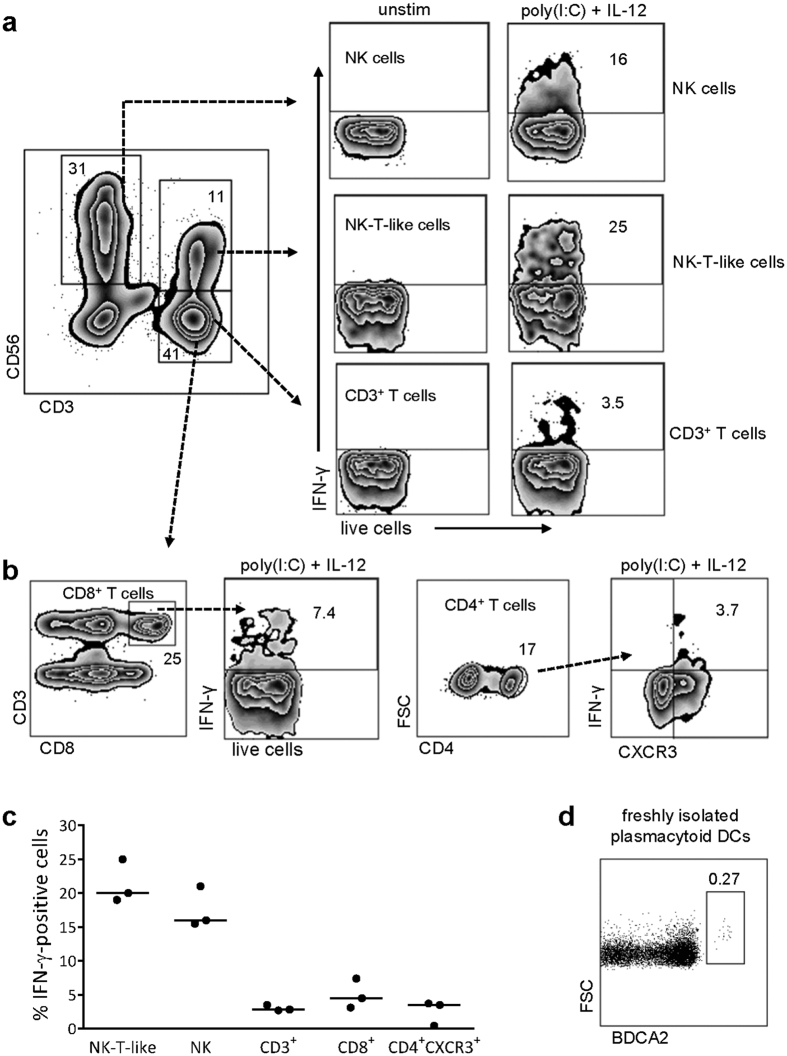
IFN-γ producing immune cells are present in decidua basalis. Mononuclear cells isolated from decidua basalis were left unstimulated or were stimulated with poly(I:C) in combination with IL-12 overnight (10^6^/ml). To identify lymphocytes, live cells were gated using a viability dye that marks all dead cells and the lymphocyte gate was then set within CD45-expressing singlet leukocytes. **(a)** Flow cytometric analysis of NK cells, NK-T-like cells and CD3^+^ T cells producing intracellular IFN-γ. **(b)** Flow cytometric analysis of CD8^+^ T cells and CXCR3^+^ CD4^+^ T cells producing intracellular IFN-γ. **(c)** The proportion of IFN-γ-positive cells among NK-T-like cells, NK cells, CD3^+^ T cells, CD8^+^ T cells and CD4^+^ CXCR3^+^ T cells (n = 3). Approximately 20.000 cells were collected in the lymphocyte gate. **(d)** Identification of freshly isolated BDCA2^+^ plasmacytoid dendritic cells among decidual mononuclear cells.

As interferon stimulation triggered BAFF secretion from decidual stromal cells, we examined if immune cells present in decidua had the ability to produce IFN-α and IFN-γ. Mononuclear cells were isolated from decidua basalis and cultured overnight with or without poly(I:C) and IL-12, and the cells were thereafter examined for intracellular cytokine production. As shown in Fig. 5a, NK cells, NK-T-like cells and CD3^+^ T cells produced intracellular IFN-γ. The highest percentage of IFN-γ producing cells was found among NK-T-like cells followed by NK cells and lastly CD3^+^ T cells (Fig. 5a,c). Among CD3^+^ T cells, a small fraction of both CD8^+^ and CXCR3^+^ CD4^+^ T cells produced IFN-γ (Fig. 5b,c). Flow cytometry results from the other two experiments are presented in Supplementary Fig. S6. It is well known that plasmacytoid dendritic cells are a major source of IFN-α. Indeed, a small population of these cells was identified among the freshly isolated decidual mononuclear cells (Fig. 5d). However, they were not detected after overnight culture, and none of the other cell subsets analyzed produced intracellular IFN-α. These results demonstrate that various immune cells competent of producing IFN-γ are present in decidua basalis that may elicit BAFF production from stromal cells.

## Discussion

BAFF is a critical cytokine for peripheral B cell maturation and is mainly produced by stromal cells^1^,^2^,^3^,^19^. We recently demonstrated that the highest BAFF levels in blood during childhood were found already at birth, and that newborns had higher levels than their mothers. Additionally, cord blood BAFF levels did not correlate with those measured in maternal blood^20^. These findings together with the fact that the origin of circulating BAFF in newborns is unknown prompted us to explore the role of decidual stromal cells as a source of BAFF. For the first time, we here demonstrate that isolated stromal cells from the maternal-derived decidual membrane secreted BAFF when stimulated with IFN-γ and IFN-α. Moreover, podoplanin was expressed in placental tissue, and also by isolated decidual stromal cells. We also show that the decidua comprised BAFF-R-expressing B cells at different maturational stages as well as IFN-γ-producing NK-T-like cells and NK cells.

Podoplanin is expressed in human placenta, mainly as a network pattern in the fetal villous stromal core^35^. In addition to this finding, we here show that podoplanin is also expressed in decidua basalis. We also found that ER-TR7 was localized in close proximity to podoplanin in the decidua and in villous core, and that neither endothelial cells, leukocytes nor epithelial cells expressed these two markers. In both human and mice, the network of podoplanin-positive T cell-zone fibroblastic reticular cells is one of the hallmarks of secondary lymphoid organs^10^,^19^. Similar structures are also present in tertiary lymphoid tissues/follicles, which are often induced at sites of chronic inflammation, e.g. in salivary glands and in the lungs of patients with Sjögrens’s syndrome or chronic obstructive pulmonary disease (COPD), respectively^10^,^15^. Interestingly, BAFF levels are increased in lungs of COPD patients and found to be expressed in close surroundings of stromal cells within the lymphoid follicles^15^. In the present study, BAFF was not detected either in decidua or in fetal villous tissue, which could be due to that BAFF is not constitutively expressed in term placentas from healthy pregnancies or to that the immunofluorescence and immunohistochemistry methods were not sensitive enough. Still, the decidua may comprise a stromal network that control feto-maternal tolerance by regulating movement and function of leukocytes during pregnancy similar to what has been shown for secondary lymphoid tissue^30^,^36^,^37^ and reviewed in ref. 38.

While most studies have focused on cross-talk between decidual stromal cells and NK cells and T cells, results from the present study indicate that stromal cells in decidua may also influence B cells. We demonstrate that isolated stromal cells from decidua basalis and decidua parietalis secreted BAFF when stimulated with IFN-γ and IFN-α, a finding corroborated by interferon-specific upregulation of BAFF mRNA levels. Although interferon-induced BAFF release from decidual stromal cells was a novel finding, these cells have previously been shown to express BAFF intracellularly in early pregnancy^25^. Furthermore, decidual stromal cells did not express BAFF-R, which suggests that these cells do not secrete BAFF in an autocrine fashion. No functional experiments with trophoblast cells were performed in the present study. It has, however, been shown that fetal villous cytotrophoblast cells do not express BAFF mRNA while mesenchymal cells isolated from the villous stroma do^39^, which correspond with podoplanin expression demonstrated in our study and by others^35^. Combined, these results indicate that placental stromal cells could be a cellular source for local BAFF and possibly also for BAFF levels in the fetal circulation. In line with this hypothesis, animal models demonstrate that non-hematopoietic stromal cells are the primary source of BAFF *in vivo*^2^,^19^. By the use of radiation chimeras, it has been shown that radiation-resistant stromal cells are the most important source of BAFF for primary B cell homeostasis^2^. More recently, a specific stromal cell population, i.e. podoplanin-positive fibroblastic reticular cells, was shown to control B cell homeostasis in lymph nodes by being the main source of local BAFF^19^.

How maternal-derived BAFF could be transported to the fetal circulation is unknown, but placental transport of certain maternal-derived proteins is generally accepted. IgG is transported from maternal to fetal circulation, which requires movement across the cytotrophoblasts, the epithelial barrier that separates the two circulations^40^,^41^. Although the mechanisms for this transport is not fully clear, there is evidence for involvement of the neonatal Fc receptor^42^. Moreover, placental amino acid transfer is likely an active process as fetal amino acid concentrations are higher than maternal concentrations^43^.

It is well established that human decidua not only comprise stromal cells but also leukocytes that belong to both innate and adaptive immunity^26^,^44^,^45^ and reviewed in ref. 46. In the present study we confirmed presence of decidual CD56^+^ NK cells, CD3^+^ T cells, CD56^+^ CD3^+^ NK-T-like cells and B cells. A novel finding, however, was that B cells at different maturational stages, i.e. immature/transitional, mature/naïve and memory cells, were present in decidua. Although a lower proportion of B cells was found among lymphocytes from decidua parietalis compared to basalis, the B cell fraction from parietalis comprised a higher percentage of memory cells than that from basalis. All decidual B cells expressed BAFF-R, which is in accordance with studies showing that BAFF-R is expressed on all transitional, mature/naïve and memory B cells in blood^47^,^48^. Thus, decidual stromal cells could be an important source of BAFF that influence maturation of B cells present in decidua during pregnancy.

The functionality of the decidual B cells was not examined here, but it has been suggested that certain B cells by their ability to secrete IL-10 possess suppressive functions that may be of importance during pregnancy. The circulating number of these cells increase in normal pregnant compared to non-pregnant women, while they are not augmented in women suffering from miscarriage^49^. Recently, the proportions of different maturational stages of circulating B cells was characterized from late pregnancy to post-partum and compared to non-pregnant women^29^. In late pregnancy, the number of B cells as well as the proportion of transitional cells was decreased compared to non-pregnant women^29^. Even though it is likely that maternal B cells are recruited to the decidua, it still remains to be elucidated how they are attracted and if various B cell subsets differ in homing properties and functions.

The cell lineage of decidual stromal cells is not fully defined, but they appear to originate from a fibroblast-like stromal cell precursor already present in the endometrium^24^,^50^. In agreement with criteria for phenotypic definition of human mesenchymal stromal cells^34^, we found that isolated decidual stromal cells expressed CD105, CD90, CD73 and CD44, and were accordingly negative for CD45. Additionally, the majority of isolated decidual stromal cells expressed podoplanin in the first passage. However, the proportion of podoplanin-positive cells clearly decreased in later passages. The reason for this can only be speculated upon, but long time culture of these cells, when no longer present in the organized decidual stromal network, might affect the expression of stromal markers. In line with this reasoning, it has been shown that ER-TR7 is expressed by lymph node stromal cells only when in contact with other cells, i.e. lymphocytes^51^. Indeed, we found that ER-TR7 was highly expressed in the decidua but not detected on the isolated stromal cells.

We also found that IFN-γ stimulation increased the number of podoplanin-positive decidual stromal cells, while the expression of BAFF-R, CD105 and ER-TR7 was unaffected. Although the significance of this finding remains to be investigated further, it has been shown that podoplanin interacts with CLEC-2, a type II transmembrane protein, primarily expressed on dendritic cells and platelets. On dendritic cells, CLEC-2 functions by facilitating efficient motility of activated dendritic cells along stromal podoplanin-expressing surfaces^13^. Thus, podoplanin upregulation in response to inflammatory stimuli may be important for regulating immune responses in the placenta during inflammation.

Several studies have shown that human innate immune cells, including macrophages, dendritic cells and neutrophils, as well as epithelial cells from different tissue locations secrete BAFF^4^,^5^,^6^,^7^,^52^, but their ability to produce physiologically relevant BAFF levels *in vivo* has not been determined. Nevertheless, it has become clear from these studies that BAFF production is triggered by stimulation with type I and II interferons, which corresponds with our results showing interferon-induced BAFF secretion from decidual stromal cells. Indeed, BAFF expression is directly downstream of type I IFN signaling and members of the IFN regulatory factor family regulate BAFF^53^. However, since neither cord blood nor maternal-derived decidual mononuclear cells produced BAFF when stimulated with either IFN-γ or IFN-α in the present study, circulating immune cells in the fetus and mother are most likely not the main source for cord blood BAFF levels.

We have previously shown that children born by mothers living on a dairy farm during pregnancy have significantly higher levels of BAFF in cord blood compared to non-farmers’ children^20^. In view of the results in the present study, one could speculate that microbes or microbial products associated with a farming environment might reach the placenta where BAFF can be produced in response to these stimuli^54^. Although the TLR agonists examined in the present study had no direct effect on BAFF secretion from stromal cells there are both innate and adaptive immune cells present in decidua, as shown in the present study and by others^26^,^44^,^45^,^46^,^56^, which may produce IFN-α and IFN-γ in response to viruses and bacteria via TLR activation that could in turn elicit BAFF production by stromal cells. Indeed, we found decidual NK-T-like cells and NK cells to be potent producers of IFN-γ in response to the viral mimic poly(I:C) combined with IL-12. CD8^+^ and CD4^+^ T cells also produced IFN-γ, albeit to a lower extent than the two former cell subsets. In line with a previous study, IFN-γ production by CD4^+^ T cells was linked to CXCR3 expression^57^. Plasmacytoid DCs were also present in the decidua, but due to the very small population of cells, IFN-α may not be as important as IFN-γ in triggering BAFF production by the decidual stromal cells in healthy pregnancies.

In several autoimmune diseases, e.g. systemic lupus erythematosus (SLE), genomic studies have shown a marked overexpression of type I interferon inducible genes that include IFN-α, the so-called IFN signature^58^. Indeed, IFN-α serum levels are higher in SLE patients compared to healthy controls^59^ and serum levels of IFN-α correlate to the validated disease activity index SLEDAI^60^. However, higher IFN-γ protein concentrations in serum have also been reported in patients with SLE compared with healthy individuals^59^. Spontaneous germinal centre (GC) responses that are associated with autoantibody production are implicated in the pathogenesis of SLE. The pathways through which GC B cell tolerance break down are not clear, but in mouse models it was recently shown that IFN-γR signaling in B cells as well as B cell IFN-γ production were critical initial steps for spontaneous GC development leading to autoimmunity^61^,^62^. Interestingly, SLE patients also display increased BAFF serum levels that correlate with disease activity^63^. In accordance with this, BAFF blockade (belimumab) is the first U.S. Food and Drug Administration-approved treatment of SLE using a biologic. Since there are reports showing an increased rate of SLE flares during pregnancy^64^,^65^, the role of the placenta as an immunological organ may be of importance to investigate further in this context.

## Additional Information

**How to cite this article:** Lundell, A-C. *et al*. IFN type I and II induce BAFF secretion from human decidual stromal cells. *Sci. Rep.* **7**, 39904; doi: 10.1038/srep39904 (2017).

**Publisher's note:** Springer Nature remains neutral with regard to jurisdictional claims in published maps and institutional affiliations.

## Supplementary Material

Supplementary Information

## Figures and Tables

**Table 1:** Antibodies used for flow cytometry, immunofluorescence and immunohistochemistry.

Flow cytometry Reactivity	Conjugate	Clone	Reference	Company
CD3	BV421	UCHT1	300434	Biolegend
CD3	FITC	SK7	345763	BD Bioscience
CD4	FITC	RPA-T4	561842	BD Bioscience
CD8	APC	RPA-T8	555369	BD Bioscience
CD19	BV421	HIB19	562441	BD Bioscience
CD20	APC-H7	L27	641414	BD Bioscience
CD24	AF647	ML5	561644	BD Bioscience
CD31	PE	WM59	555446	BD Bioscience
CD38	PE	HB-7	345806	BD Bioscience
CD56	PE	HCD56	318306	Biolegend
CD56	BV421	HCD56	318328	Biolegend
CD14/45	PE/FITC	MφP9/2D1	342408	BD Bioscience
CD44	PE	G44-26	555478	BD Bioscience
CD45	PerCP-Cy5.5	HI30	564105	BD Bioscience
CD73	FITC	AD2	550257	BD Bioscience
CD90	FITC	5E10	329706	Biolegend
CD105	AF647	266	561439	BD Bioscience
CD105	PE	266	560839	BD Bioscience
CD303 (BDCA2)	APC	AC144	130-097-931	Miltenyi Biotec
BAFF-R	FITC	8A7	11-9117-42	eBioscience
CXCR3	BV421	G025H7	353716	Biolegend
IFN-γ	PE	B27	562016	BD Bioscience
IFN-α	PE	LT27:295	130-099-098	Miltenyi Biotec
**Flow cytometry, Immunofluorescence and Immunohistochemistry**				
**Primary antibodies Reactivity**	**Isotype**	**Clone**	**Reference**	**Company**
Podoplanin	Mouse IgG1	D2-40	MCA2543	Serotec
ER-TR7	Rat IgG2a	ER-TR7	Sc-73355	Santa Cruz
CD45	Mouse IgG1	HI30	564105	BD Bioscience
CD31	Mouse IgG1	WM59	555446	BD Bioscience
**Flow cytometry Secondary antibodies Reactivity**	**Host/Isotype**	**Conjugate**	**Reference**	**Company**
Mouse IgG	Rabbit Ig	Biotin	E0464	DAKO
Streptavidin		BV421	405226	Biolegend
Rat IgG	Goat IgG	AF488	A11006	Thermo Fisher Scientific
**Immunofluorescence Secondary antibodies Reactivity**				
Mouse IgG	Rabbit IgG	AF488	A11059	Thermo Fisher Scientific
Rat IgG	Goat IgG	AF555	A21434	Thermo Fisher Scientific

## References

[b1] Schiemann B. . An essential role for BAFF in the normal development of B cells through a BCMA-independent pathway. Science 293, 2111–2114 (2001). 11509691 10.1126/science.1061964

[b2] Gorelik L. . Normal B cell homeostasis requires B cell activation factor production by radiation-resistant cells. J Exp Med 198, 937–945 (2003). 12975458 10.1084/jem.20030789 PMC2194202

[b3] Warnatz K. . B-cell activating factor receptor deficiency is associated with an adult-onset antibody deficiency syndrome in humans. Proc Natl Acad USA 106, 13945–13950 (2009). 10.1073/pnas.0903543106 PMC2722504 19666484

[b4] Nardelli B. . Synthesis and release of B-lymphocyte stimulator from myeloid cells. Blood 97, 198–204 (2001). 11133761 10.1182/blood.v97.1.198

[b5] Scapini P. . G-CSF-stimulated neutrophils are a prominent source of functional BLyS. J Exp Med 197, 297–302 (2003). 12566413 10.1084/jem.20021343 PMC2193843

[b6] Ittah M. . B cell-activating factor of the tumor necrosis factor family (BAFF) is expressed under stimulation by interferon in salivary gland epithelial cells in primary Sjogren’s syndrome. Arthritis Res & Ther 8, R51 (2006). 16507175 10.1186/ar1912 PMC1526588

[b7] Woo S. J. . Induction of BAFF expression by IFN-gamma via JAK/STAT signaling pathways in human intestinal epithelial cells. J Leuk Biol 93, 363–368 (2013). 10.1189/jlb.0412210 23271704

[b8] Link A. . Fibroblastic reticular cells in lymph nodes regulate the homeostasis of naive T cells. Nature Immunol 8, 1255–1265 (2007). 17893676 10.1038/ni1513

[b9] Broggi M. A., Schmaler M., Lagarde N. & Rossi S. W. Isolation of murine lymph node stromal cells. J Vis Exp, e51803 (2014). 25178108 10.3791/51803 PMC4827973

[b10] Link A. . Association of T-zone reticular networks and conduits with ectopic lymphoid tissues in mice and humans. Am J Pathol 178, 1662–1675 (2011). 21435450 10.1016/j.ajpath.2010.12.039 PMC3070229

[b11] Schacht V. . T1alpha/podoplanin deficiency disrupts normal lymphatic vasculature formation and causes lymphedema. EMBO J 22, 3546–3556 (2003). 12853470 10.1093/emboj/cdg342 PMC165612

[b12] Suzuki-Inoue K. . Involvement of the snake toxin receptor CLEC-2, in podoplanin-mediated platelet activation, by cancer cells. J Biol Chem 282, 25993–26001 (2007). 17616532 10.1074/jbc.M702327200

[b13] Acton S. E. . Podoplanin-rich stromal networks induce dendritic cell motility via activation of the C-type lectin receptor CLEC-2. Immunity 37, 276–289 (2012). 22884313 10.1016/j.immuni.2012.05.022 PMC3556784

[b14] Katakai T., Hara T., Sugai M., Gonda H. & Shimizu A. Lymph node fibroblastic reticular cells construct the stromal reticulum via contact with lymphocytes. J Exp Med 200, 783–795 (2004). 15381731 10.1084/jem.20040254 PMC2211971

[b15] Seys L. J. . Role of B Cell-Activating Factor in Chronic Obstructive Pulmonary Disease. Am J Resp Crit Med 192, 706–718 (2015). 10.1164/rccm.201501-0103OC 26266827

[b16] Bajenoff M. . Stromal cell networks regulate lymphocyte entry, migration, and territoriality in lymph nodes. Immunity 25, 989–1001 (2006). 17112751 10.1016/j.immuni.2006.10.011 PMC2692293

[b17] Denton A. E., Roberts E. W., Linterman M. A. & Fearon D. T. Fibroblastic reticular cells of the lymph node are required for retention of resting but not activated CD8+ T cells. Proc Natl Acad Sci USA 111, 12139–12144 (2014). 25092322 10.1073/pnas.1412910111 PMC4143042

[b18] Astarita J. L. . The CLEC-2-podoplanin axis controls the contractility of fibroblastic reticular cells and lymph node microarchitecture. Nature Immunol 16, 75–84 (2015). 25347465 10.1038/ni.3035 PMC4270928

[b19] Cremasco V. . B cell homeostasis and follicle confines are governed by fibroblastic reticular cells. Nature Immunol 15, 973–981 (2014). 25151489 10.1038/ni.2965 PMC4205585

[b20] Lundell A. C. . Higher B-cell activating factor levels at birth are positively associated with maternal dairy farm exposure and negatively related to allergy development. J Allergy Clin Immunol 136, 1074–1082 e1073 (2015). 25936566 10.1016/j.jaci.2015.03.022

[b21] Kreuzaler M. . Soluble BAFF levels inversely correlate with peripheral B cell numbers and the expression of BAFF receptors. J Immunol 188, 497–503 (2012). 22124120 10.4049/jimmunol.1102321

[b22] Bienertova-Vasku J. . The presence of B-cell activating factor (BAFF) in umbilical cord blood in both healthy and pre-eclamptic pregnancies and in human breast milk. J Reprod Immunol 109, 89–93 (2015). 25656062 10.1016/j.jri.2014.12.003

[b23] Sindram-Trujillo A., Scherjon S., Kanhai H., Roelen D. & Claas F. Increased T-cell activation in decidua parietalis compared to decidua basalis in uncomplicated human term pregnancy. Am J Reprod Immunol 49, 261–268 (2003). 12854730 10.1034/j.1600-0897.2003.00041.x

[b24] Richards R. G., Brar A. K., Frank G. R., Hartman S. M. & Jikihara H. Fibroblast cells from term human decidua closely resemble endometrial stromal cells: induction of prolactin and insulin-like growth factor binding protein-1 expression. Biol Reprod 52, 609–615 (1995). 7756454 10.1095/biolreprod52.3.609

[b25] Munoz-Fernandez R. . Human decidual stromal cells secrete C-X-C motif chemokine 13, express B cell-activating factor and rescue B lymphocytes from apoptosis: distinctive characteristics of follicular dendritic cells. Hum Reprod 27, 2775–2784 (2012). 22718279 10.1093/humrep/des198

[b26] Xu Y., Plazyo O., Romero R., Hassan S. S. & Gomez-Lopez N. Isolation of Leukocytes from the Human Maternal-fetal Interface. J Vis Exp, e52863 (2015). 26067211 10.3791/52863 PMC4467471

[b27] Haller H. . An immunohistochemical study of leucocytes in human endometrium, first and third trimester basal decidua. J Reprod Immunol 23, 41–49 (1993). 8429523 10.1016/0165-0378(93)90025-d

[b28] Gomez-Lopez N., Vadillo-Perez L., Nessim S., Olson D. M. & Vadillo-Ortega F. Choriodecidua and amnion exhibit selective leukocyte chemotaxis during term human labor. Am J Obstet Gynecol 204, 364 e369–316 (2011). 10.1016/j.ajog.2010.11.010 21296334

[b29] Lima J. . Characterization of B cells in healthy pregnant women from late pregnancy to post-partum: a prospective observational study. BMC Pregnancy Chilsbirth 16, 139 (2016). 10.1186/s12884-016-0927-7 PMC4895979 27267973

[b30] Karlsson H. . Stromal cells from term fetal membrane are highly suppressive in allogeneic settings *in vitro*. Clin Exp Immunol 167, 543–555 (2012). 22288598 10.1111/j.1365-2249.2011.04540.x PMC3374287

[b31] Ringden O. . Fetal membrane cells for treatment of steroid-refractory acute graft-versus-host disease. Stem Cells 31, 592–601 (2013). 23307526 10.1002/stem.1314

[b32] Leno-Duran E. . Human decidual stromal cells secrete soluble pro-apoptotic factors during decidualization in a cAMP-dependent manner. Hum Reprod 29, 2269–2277 (2014). 25124667 10.1093/humrep/deu202

[b33] Greaves R. F. . Hormone modeling in preterm neonates: establishment of pituitary and steroid hormone reference intervals. J Clin Endocrinol Metab 100, 1097–1103 (2015). 25562509 10.1210/jc.2014-3681

[b34] Dominici M. . Minimal criteria for defining multipotent mesenchymal stromal cells. The International Society for Cellular Therapy position statement. Cytotherapy 8, 315–317 (2006). 16923606 10.1080/14653240600855905

[b35] Wang Y. . D2-40/podoplanin expression in the human placenta. Placenta 32, 27–32 (2011). 21095001 10.1016/j.placenta.2010.10.014 PMC3062260

[b36] Croxatto D. . Stromal cells from human decidua exert a strong inhibitory effect on NK cell function and dendritic cell differentiation. PloS one 9, e89006 (2014). 24586479 10.1371/journal.pone.0089006 PMC3930605

[b37] Liu W. . Human placenta-derived adherent cells induce tolerogenic immune responses. Clin Transl Immunol 3, e14 (2014). 10.1038/cti.2014.5 PMC4232071 25505962

[b38] Chang J. E. & Turley S. J. Stromal infrastructure of the lymph node and coordination of immunity. Trend Immunol 36, 30–39 (2015). 10.1016/j.it.2014.11.003 25499856

[b39] Langat D. L., Wheaton D. A., Platt J. S., Sifers T. & Hunt J. S. Signaling pathways for B cell-activating factor (BAFF) and a proliferation-inducing ligand (APRIL) in human placenta. Am J Pathol 172, 1303–1311 (2008). 18403603 10.2353/ajpath.2008.071139 PMC2329839

[b40] Fuchs R. & Ellinger I. Endocytic and transcytotic processes in villous syncytiotrophoblast: role in nutrient transport to the human fetus. Traffic 5, 725–738 (2004). 15355509 10.1111/j.1600-0854.2004.00221.x

[b41] Cleal J. K. . Facilitated transporters mediate net efflux of amino acids to the fetus across the basal membrane of the placental syncytiotrophoblast. J Physiol 589, 987–997 (2011). 21224231 10.1113/jphysiol.2010.198549 PMC3060375

[b42] Simister N. E. Placental transport of immunoglobulin G. Vaccine 21, 3365–3369 (2003). 12850341 10.1016/s0264-410x(03)00334-7

[b43] Cetin I. . Maternal and fetal amino acid concentrations in normal pregnancies and in pregnancies with gestational diabetes mellitus. Am J Obstet Gynecol 192, 610–617 (2005). 15696011 10.1016/j.ajog.2004.08.011

[b44] Koopman L. A. . Human decidual natural killer cells are a unique NK cell subset with immunomodulatory potential. J Exp Med 198, 1201–1212 (2003). 14568979 10.1084/jem.20030305 PMC2194228

[b45] Heikkinen J., Mottonen M., Komi J., Alanen A. & Lassila O. Phenotypic characterization of human decidual macrophages. Clin Exp Immunol 131, 498–505 (2003). 12605704 10.1046/j.1365-2249.2003.02092.x PMC1808648

[b46] Erlebacher A. Immunology of the maternal-fetal interface. Ann Rev Immunol 31, 387–411 (2013). 23298207 10.1146/annurev-immunol-032712-100003

[b47] Cuss A. K. . Expansion of functionally immature transitional B cells is associated with human-immunodeficient states characterized by impaired humoral immunity. J Immunol 176, 1506–1516 (2006). 16424179 10.4049/jimmunol.176.3.1506

[b48] Palanichamy A. . Novel human transitional B cell populations revealed by B cell depletion therapy. J Immunol 182, 5982–5993 (2009). 19414749 10.4049/jimmunol.0801859 PMC2746373

[b49] Rolle L. . Cutting edge: IL-10-producing regulatory B cells in early human pregnancy. Am J Reprod Immunol 70, 448–453 (2013). 24118333 10.1111/aji.12157

[b50] Oliver C., Montes M. J., Galindo J. A., Ruiz C. & Olivares E. G. Human decidual stromal cells express alpha-smooth muscle actin and show ultrastructural similarities with myofibroblasts. Hum Reprod 14, 1599–1605 (1999). 10357983 10.1093/humrep/14.6.1599

[b51] Katakai T. Marginal reticular cells: a stromal subset directly descended from the lymphoid tissue organizer. Front Immunol 3, 200 (2012). 22807928 10.3389/fimmu.2012.00200 PMC3395019

[b52] Kato A., Truong-Tran A. Q., Scott A. L., Matsumoto K. & Schleimer R. P. Airway epithelial cells produce B cell-activating factor of TNF family by an IFN-beta-dependent mechanism. J Immunol 177, 7164–7172 (2006). 17082634 10.4049/jimmunol.177.10.7164 PMC2804942

[b53] Sjostrand M. . The Expression of BAFF Is Controlled by IRF Transcription Factors. J Immunol (2015). 10.4049/jimmunol.1501061 PMC4683359 26590315

[b54] Aagaard K. . The placenta harbors a unique microbiome. Sci Trans Med 6, 237ra265 (2014). 10.1126/scitranslmed.3008599 PMC4929217 24848255

[b55] Stout M. J. . Identification of intracellular bacteria in the basal plate of the human placenta in term and preterm gestations. Am J Obstet Gynecol 208, 226 e221–227 (2013). 10.1016/j.ajog.2013.01.018 PMC3740162 23333552

[b56] Ban Y. L., Kong B. H., Qu X., Yang Q. F. & Ma Y. Y. BDCA-1+, BDCA-2+ and BDCA-3+ dendritic cells in early human pregnancy decidua. Clin Exp Immunol 151, 399–406 (2008). 18234052 10.1111/j.1365-2249.2007.03576.x PMC2276959

[b57] Pandya J. M. . Circulating T helper and T regulatory subsets in untreated early rheumatoid arthritis and healthy control subjects. J Leuk Biol 100, 823–833 (2016). 10.1189/jlb.5A0116-025R 27190305

[b58] Higgs B. W. . Patients with systemic lupus erythematosus, myositis, rheumatoid arthritis and scleroderma share activation of a common type I interferon pathway. Ann Rheum Dis 70, 2029–2036 (2011). 21803750 10.1136/ard.2011.150326

[b59] Kim T. . Serum levels of interferons in patients with systemic lupus erythematosus. Clin Exp Immunol 70, 562–569 (1987). 2449306 PMC1542177

[b60] Bengtsson A. A. . Activation of type I interferon system in systemic lupus erythematosus correlates with disease activity but not with antiretroviral antibodies. Lupus 9, 664–671 (2000). 11199920 10.1191/096120300674499064

[b61] Domeier P. P. . IFN-gamma receptor and STAT1 signaling in B cells are central to spontaneous germinal center formation and autoimmunity. J Exp Med 213, 715–732 (2016). 27069112 10.1084/jem.20151722 PMC4854731

[b62] Jackson S. W. . B cell IFN-gamma receptor signaling promotes autoimmune germinal centers via cell-intrinsic induction of BCL-6. J Exp Med 213, 733–750 (2016). 27069113 10.1084/jem.20151724 PMC4854732

[b63] Salazar-Camarena D. C. . Association of BAFF, APRIL serum levels, BAFF-R, TACI and BCMA expression on peripheral B-cell subsets with clinical manifestations in systemic lupus erythematosus. Lupus (2015). 10.1177/0961203315608254 26424128

[b64] Petri M., Howard D. & Repke J. Frequency of lupus flare in pregnancy. The Hopkins Lupus Pregnancy Center experience. Arthritis Rheum 34, 1538–1545 (1991). 1670196 10.1002/art.1780341210

[b65] Ruiz-Irastorza G. . Increased rate of lupus flare during pregnancy and the puerperium: a prospective study of 78 pregnancies. Br J Rheum 35, 133–138 (1996). 10.1093/rheumatology/35.2.133 8612024

