# Supplementary material for: IFN type I and II induce BAFF secretion from human decidual stromal cells
Source: Sci Rep. 2017 Jan 6;7:39904. doi: 10.1038/srep39904 (PMC5216379; doi:10.1038/srep39904)
Supplement: Supplementary Information [file srep39904-s1.pdf]

IFN type I and II induce BAFF secretion from human decidual stromal cells

Anna-Carin Lundell[1*], Inger Nordström[1], Kerstin Andersson[1], Christina Lundqvist[1], Esbjörn telemo[1], Silvia Nava[2], Helen Kaipe[2,3] and Anna Rudin[1]

Supplementary Figure S1

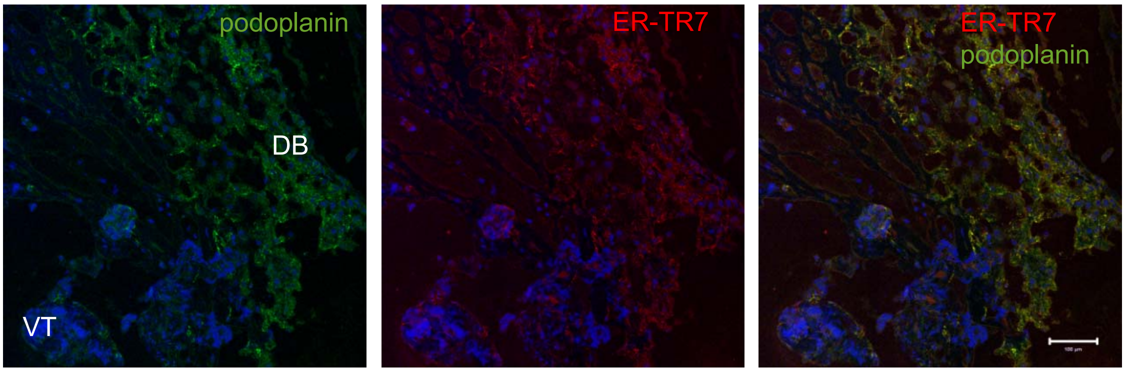

Supplementary Figure S1. Podoplanin and ER-TR7 expression in placental tissue. Immunofluorescence staining of podoplanin (green), ER-TR7 (red) and merge of the two markers (yellow) in decidua basalis (DB) and in the underlying villous tissue (VT). Nuclei staining with Hoechst in blue (x25 magnification).

Supplementary Figure S2

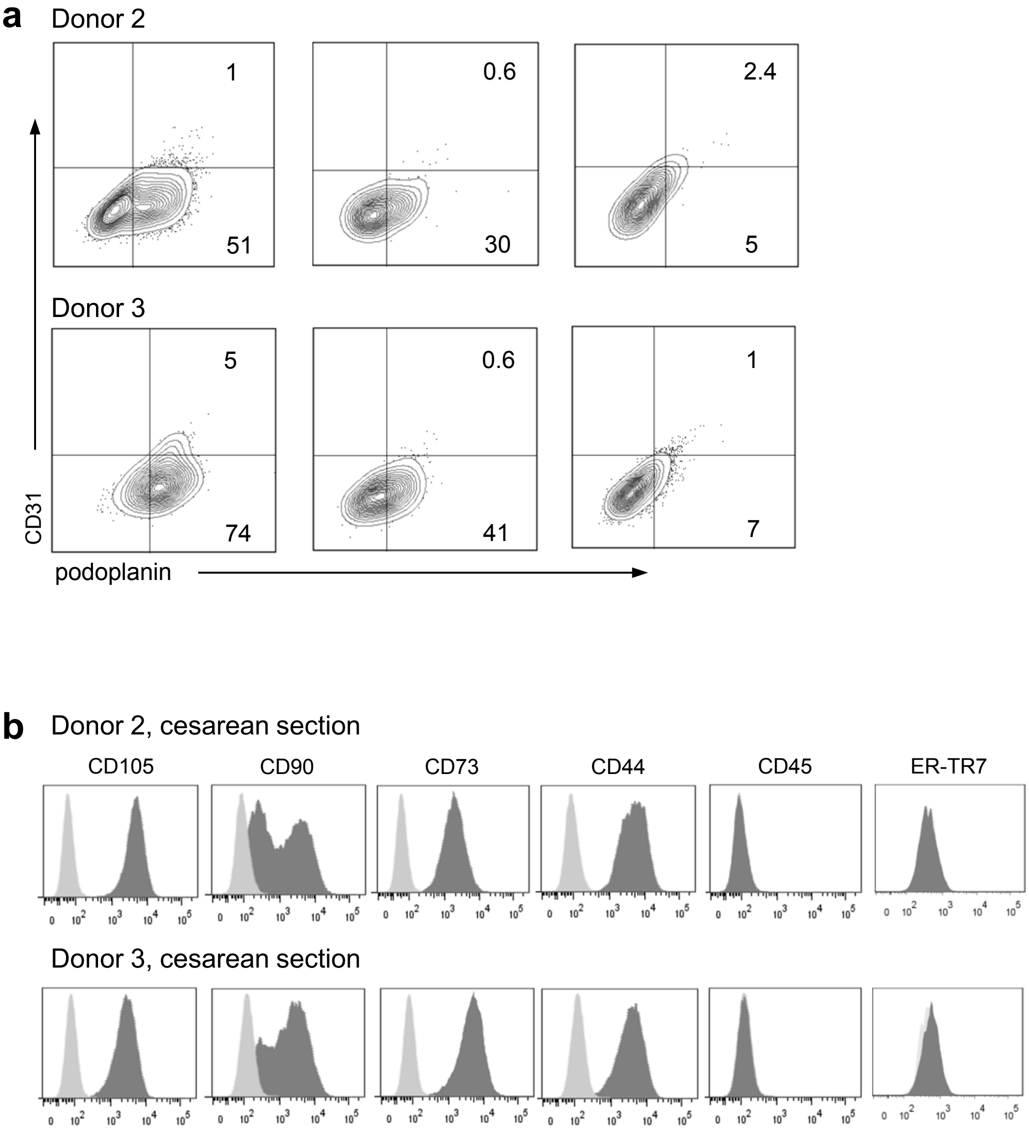

Supplementary Figure S2. Phenotypic characterization of decidual stromal cells.
(a) Expression of podoplanin and CD31 on decidual stromal cells at different passages.
(b) Expression of characteristic stroma cell markers on cells isolated from decidua basalis after cesarean section. Specific markers are depicted in dark grey and FMO controls in light grey.

Supplementary Figure S3

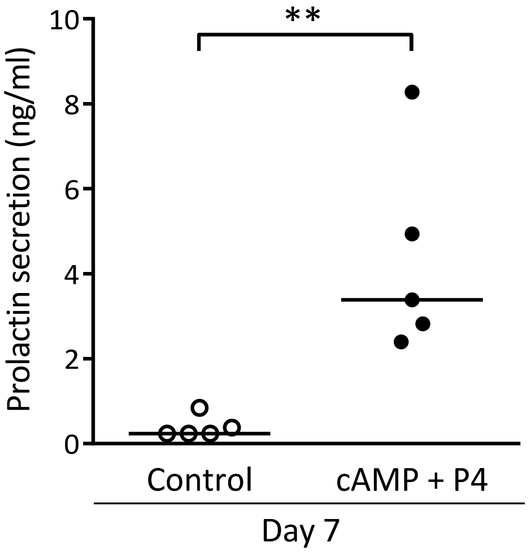

Supplementary Figure S3. Decidualized stromal cells secrete prolactin. Prolactin secretion by stromal cells from 5 different donors cultured in the presence of progesterone (P4) and cAMP for 7 days. Horizontal bars indicate median. **P≤0.01 Mann-Whitney U test.

Supplementary Figure S4

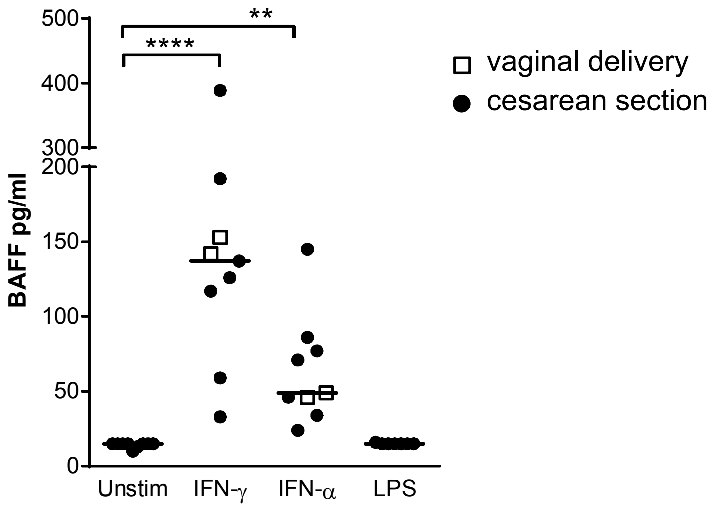

Supplementary Figure S4. BAFF secretion by decidual stromal cells in relation to delivery mode. BAFF secretion by isolated decidual stromal cells in response to IFN-γ, IFN-α or LPS. The open squares indicate BAFF secretion from decidual stromal cells isolated after vaginal delivery and filled circles cesarean section. Horizontal bars indicate median. **P≤0.01 and ****P≤0.0001, Kruskal-Wallis test followed by Dunn's multiple comparison test.

Supplementary Figure S5

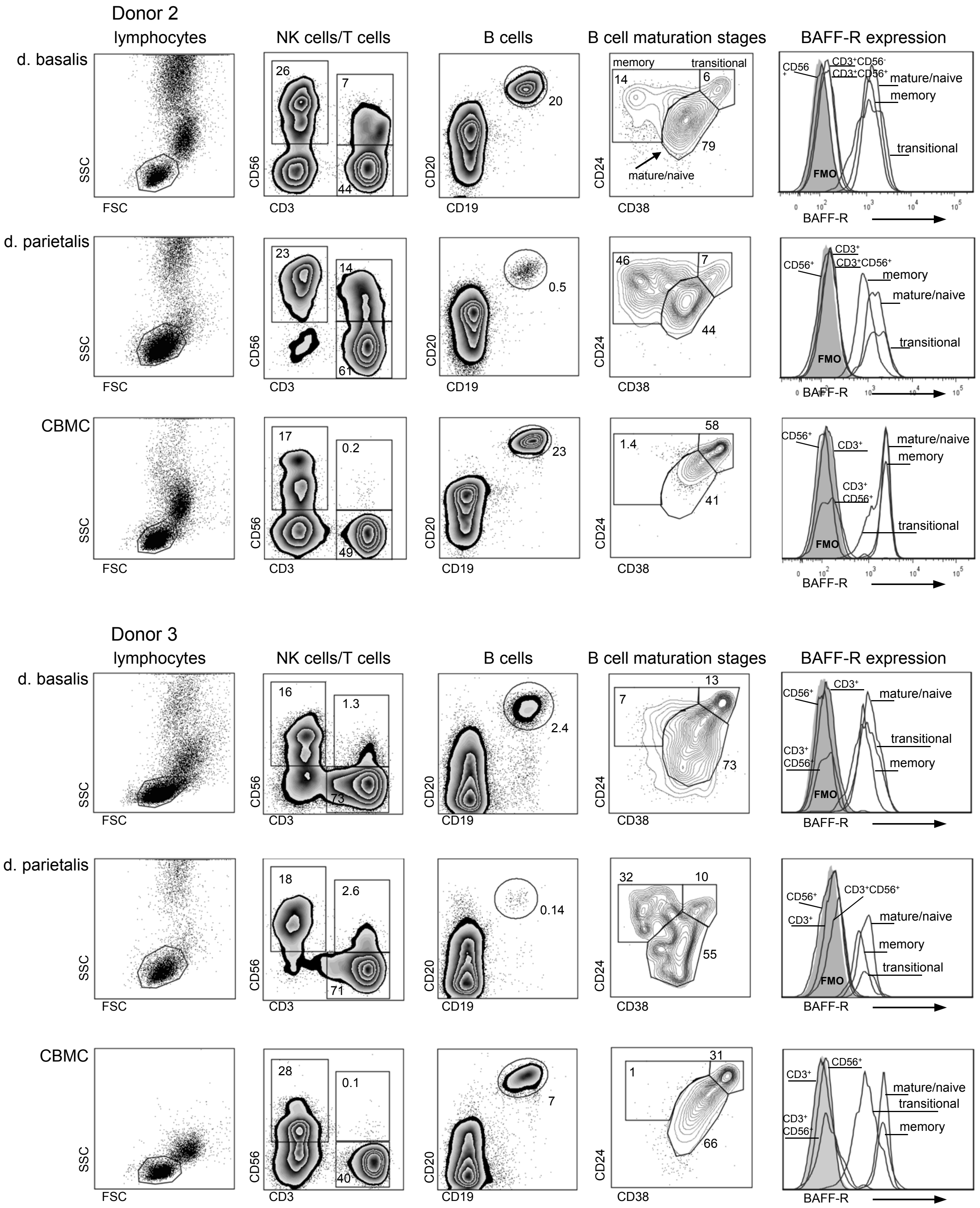

Supplementary Figure S5. BAFF-R expressing B cells at different maturational stages in decidua.
To identify lymphocytes, in the first panel to the far left, live cells were gated using a viability dye and lymphocyte gate was then set within CD45-positive singlet cells. Within lymphocytes, $CD56^{+}$ NK cells, $CD3^{+}CD56^{neg}$ T cells, $CD3^{+}CD56^{+}$ NK-T-like cells and B cells were identified (second and third panels). In the fourth panel, B cell maturational stages were analyzed. In the panel to the far right, BAFF-R expression on the different lymphocyte populations. Approximately 40.000 cells were collected in the lymphocyte gate for d. basalis and CBMC and 20.000 for d. parietalis.

Supplementary Figure S6

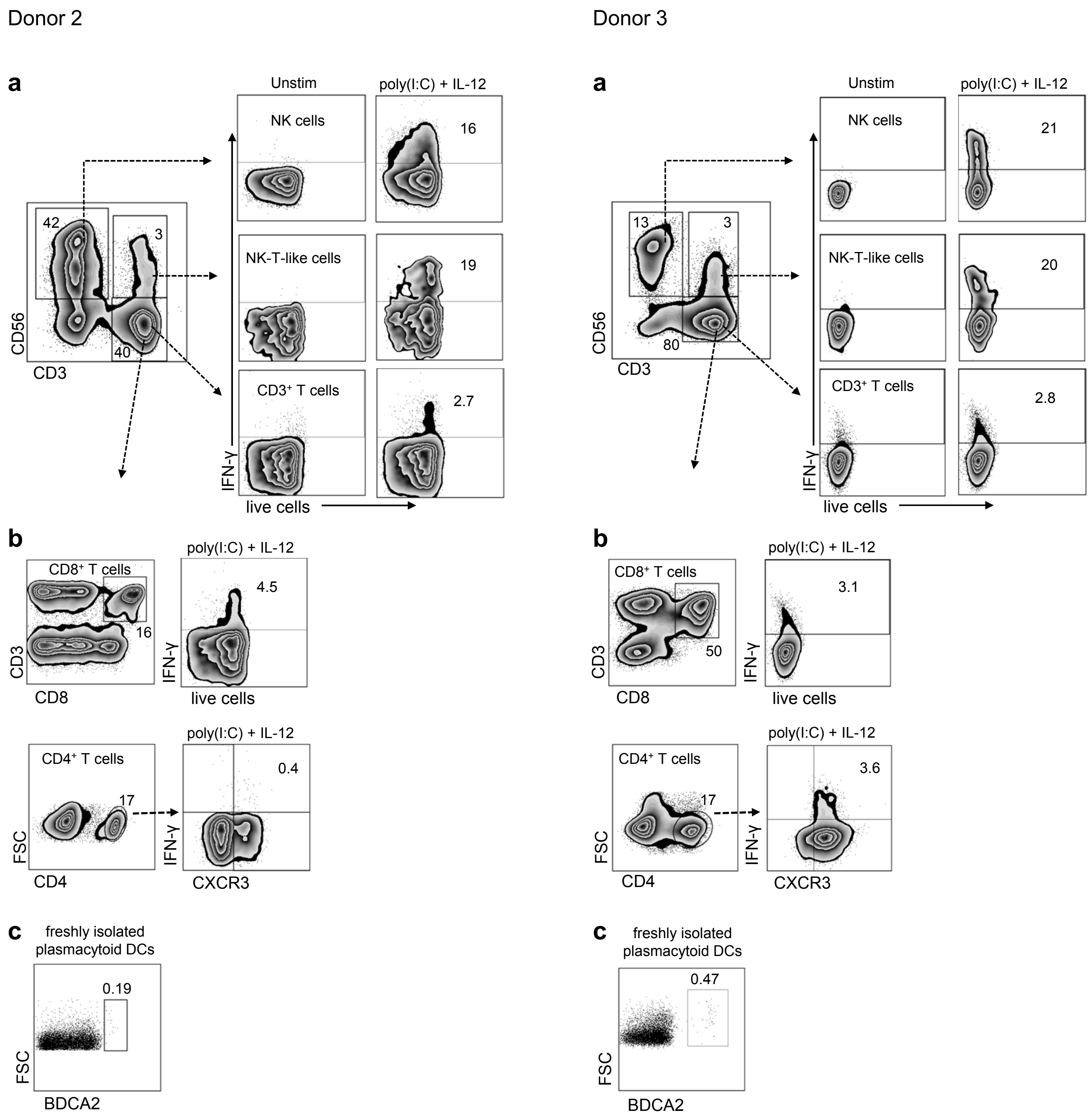

Supplementary Figure S6. IFN-γ producing immune cells in decidua basalis. Mononuclear cells isolated from decidua basalis were left unstimulated or were stimulated with poly(I:C) and IL-12 overnight. To identify lymphocytes, live cells were gated using a viability dye and lymphocyte gate was then set within CD45-expressing singlet leukocytes. About 20.000 cells were collected in the lymphocyte gate. **(a)** Analysis of NK cells, NK-T-like cells and T cells producing intracellular IFN-γ. **(b)** Analysis of $CD8^+$ T cells and $CXCR3^+CD4^+$ T cells producing intracellular IFN-γ. Approximately 20.000 cells were collected in the lymphocyte gate. **(c)** Identification of freshly isolated $BDCA2^+$ plasmacytoid dendritic cells among decidual mononuclear cells.
